# Successful restoration of failed Achilles tendon surgery with semitendinosus and gracilis tendon graft: a case report

**DOI:** 10.3389/fsurg.2023.1233502

**Published:** 2023-11-20

**Authors:** Yu-Tun Hung, Elaheh Alizargar, Javad Alizargar, Kun-Chin Hung, Chien-Min Chen, Ru-Yu Pan

**Affiliations:** ^1^ Department of Medicine, Hualien Armed Forces General Hospital, Hualien, Taiwan; ^2^ Department of Orthopedics, Tri-Service General Hospital, National Defense Medical Center, Taipei, Taiwan; ^3^ Institute of Public Health, National Yang Ming Chiao Tung University, Taipei, Taiwan; ^4^ School of Nursing, National Taipei University of Nursing and Health Sciences, Taipei, Taiwan; ^5^ Department of Medicine, Kwan Hua Hospital, Changhua, Taiwan; ^6^ Department of Neurosurgery, Changhua Christian Hospital, Changhua, Taiwan

**Keywords:** Achilles tendon, partial tear, surgery, gracilis tendon graft, semitendinosus tendon graft

## Abstract

**Objective:**

This case presentation aims to highlight the challenges and outcomes associated with a partial tear of the Achilles tendon (AT) in an elite marathon runner. The objective was to restore tendon anatomy and optimize strength recovery through surgical intervention.

**Method:**

We present the case of a marathon runner who suffered a partial AT tear and underwent an initial surgery that failed. A revision surgery was then performed using a semitendinosus and gracilis tendon graft.

**Results:**

The patient underwent surgery 14 weeks after the initial AT injury, but unfortunately experienced a complete AT tear after 6 months. However, the novel aspect of this case is the successful restoration of the failed double-row suture technique through the utilization of a semitendinosus and gracilis tendon graft. Notably, the graft remained intact even under high tendon loading during the 2-year follow-up period.

**Conclusion:**

Differential diagnosis should consider partial tears of the AT as a possible diagnosis in the patients with heel pain. Furthermore, it is crucial to prioritize a well-paced post-surgical rehabilitation process in AT surgeries. In cases of failed AT surgery, the utilization of gracilis and semitendinosus tendon grafts can serve as viable options for repairing reruptures.

## 1. Introduction

A partial tear of the Achilles Tendon (AT) is a rare condition in which there is a partial discontinuation of the tendon. It usually occurs suddenly and causes pain during loading and a feeling of weakness. Athletes suffering from a partial tear may still be able to train but cannot reach maximal loading. Physically active patients may experience permanent symptoms or initial warm-up pain after a period of resting, which may increase or decrease during physical activity. Clinical findings include tendon thickening, localized pain, palpable tendon discontinuity, loss of function, and limping (1). Lemme et al. reported the highest incidence of AT rupture was in males aged 20–39 years and in females aged 40–59 years. The most common cause of AT ruptures was participation in sports or recreational activities, with basketball being the most common overall cause (2).

Elite athletes who sustain AT injuries often opt for surgery to restore tendon anatomy and optimize strength recovery. However, postoperative care and rehabilitation require extended time off from play, potentially affecting postoperative athletic performance. Return-to-play (RTP) guidelines (3) suggest 16 weeks for non-contact athletes and 20 weeks for contact athletes. While most elite athletes with AT ruptures can return to their pre-injury level, the impact on performance varies. NBA studies showed a significant decline in performance, while NFL, NCAA football, and professional soccer studies reported mixed results. MLB players did not experience significant performance decreases. It remains unclear if these performance effects persist as athletes progress in their careers.

The diagnosis of an AT injury is typically established by considering the patient's history, which involves the sudden onset of pain and difficulty fully loading the tendon. Prior intratendinous cortisone injections may also be part of the patient's medical history. Clinical examination often reveals localized tenderness in the tendon area and weakness during heel raises. Ultrasound and Doppler exams can show an irregular and bulging superficial tendon line, along with localized increased blood flow. Magnetic resonance imaging (MRI) typically demonstrates a hyperintense signal in the tendon on both T1 and T2-weighted sequences.

The initial treatment approach for AT injuries involves conservative measures. This includes the use of a 2 cm heel lift for the first 6 weeks to reduce tension on the tendon and avoiding stretching the tendon for 12 weeks. Subsequently, a reduced heel lift of 1 cm is utilized, and progressive loading of the tendon is initiated between weeks 7-12. If there is no pain, the heel lift can be removed, and eccentric exercises can be gradually introduced. In cases where conservative management is ineffective, surgical intervention may be necessary. Surgical options may involve exploration, excision of the partial rupture, and suturing. For partial ruptures near the Achilles insertion, augmentation procedures or anchor applications may be considered based on the size and location of the injury. Following surgery, a rehabilitation program lasting 12–14 weeks is recommended before resuming full tendon-loading activities (1).

In our case presentation, we describe a marathon runner who experienced a partial AT tear that underwent a surgery which failed at the first and a successful revision surgery using a semitendinosus and gracilis tendon graft.

## 2. Case description

A 49-year-old male amateur marathon runner presented with swelling, tenderness, and unilateral heel pain in his left AT, which had worsened over the past month. The patient had previously experienced chronic AT pain for several months, including numbness in his left heel while running, jumping, and walking down the stairs. At the onset of symptoms, AT function was preserved, and the Thompson test was negative, but the patient had soreness and a burning sensation. The patient had no history of taking analgesic drugs despite the chronic pain.

Before coming to our clinic, he received a steroid injection in another medical facility to alleviate his symptoms. He had a medical history of septoplasty for nasal septum deviation 20 years ago, but he was otherwise healthy, physically active, and had no history of smoking, alcohol, or substance abuse. He did not recall any history of trauma such as a collision or sprain injury to his left heel.

**Figure 1:**
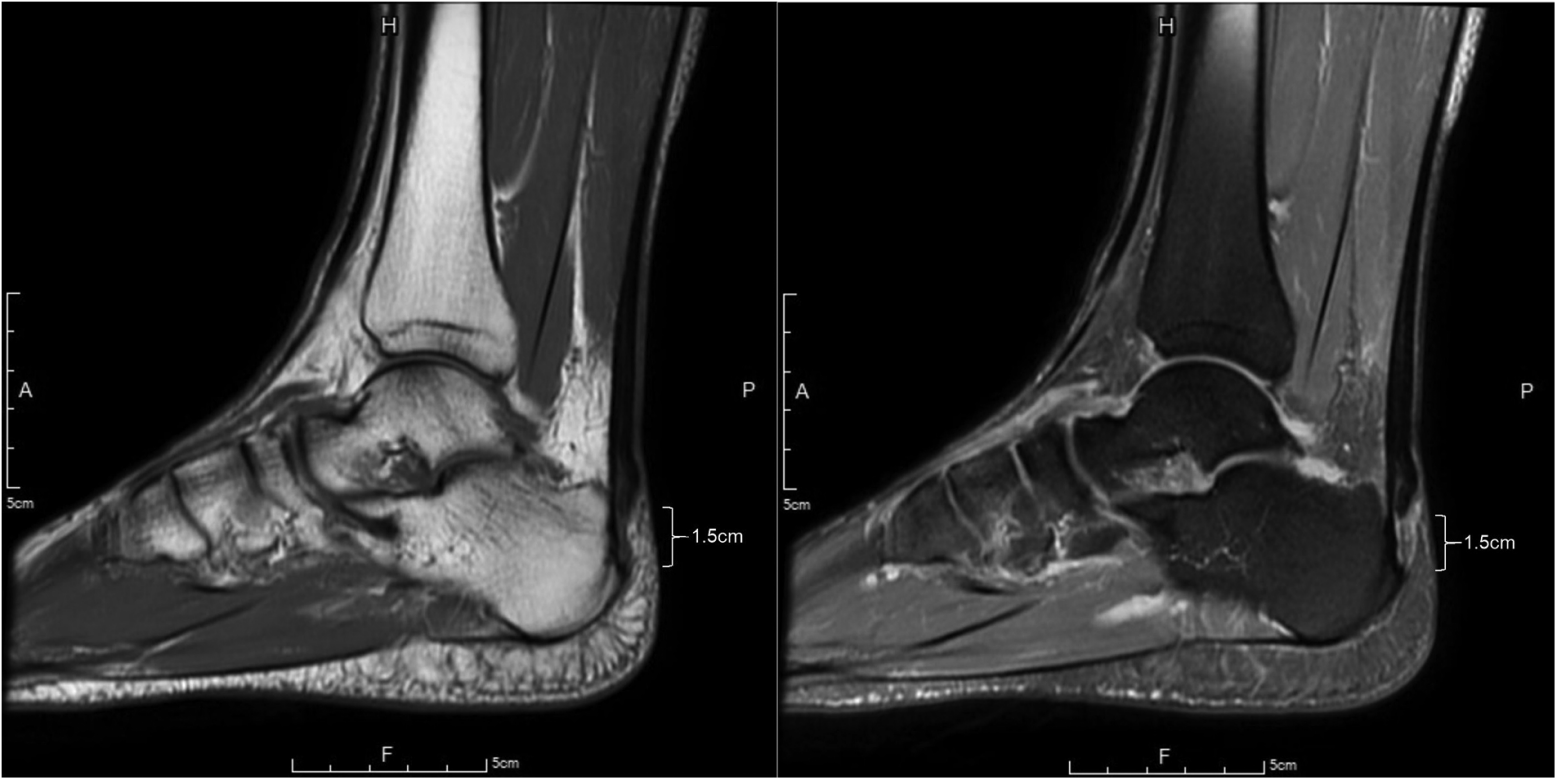
MRI images of the patient's Achilles tendon. Mid-sagittal view using T1-weighted imaging (left) and sagittal view using PD-FS imaging (right).

The patient underwent an MRI which revealed a partial tear of more than 50% of the left AT with posterior proximal segment retraction of about 1.5 cm. The Figure 1 shows two different MRI images of the patient's AT. The first image (left) is a mid-sagittal view using T1-weighted imaging and reveals a partial-thickness tear of the tendon extending approximately 1.5 cm from its point of insertion at the calcaneus. The second image (right) is a sagittal view using PD-FS imaging and displays inflammation of the enthuses, along with an increase in mucoid, proteoglycan, and water content. This finding is consistent with subcutaneous bursitis, which is commonly seen in insertional Achilles tendinopathy. The patient was given the option of operative or non-operative management, and he chose the operative management due to his desire to participate in a marathon.

### 2.1. First operation

During the first operation, the proximal AT stump was found to be mobile and able to move around a finger. The ruptured ends of the tendon were matched to achieve the optimal length using non-traumatic clamps. The tendon edges were then approximated and sutured together. A rectangular flap, measuring 1–2 cm in width and 8 cm in length, was taken from the proximal tendon and gastrocnemius aponeurosis, and pulled down by approximately 3 cm beyond the rupture site. This flap was sutured down, utilizing the double row transosseous-equivalent (TOE) bridge suture method and involved the use of four 4.75 mm Arthex Swive-Lock suture tape anchors. After the surgery, a short leg splint was applied with the ankle in plantar flexion.

**Figure 2:**
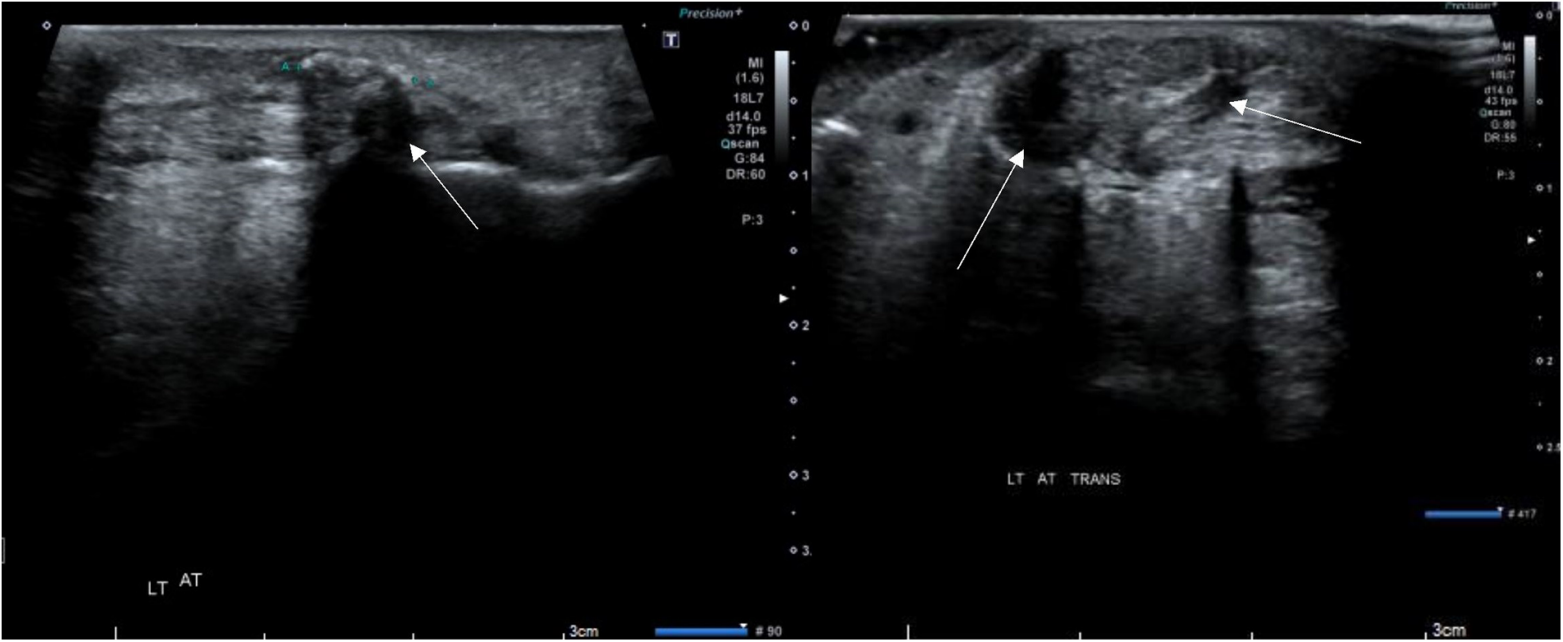
Ultrasonography images of the patient's AT after the first surgery. The sagittal image (left) shows an increase in mucoid and fluid content, indicating post-operative inflammation. The transverse image (right) displays fluid accumulation surrounding the AT, with bright spots enhancing the image.

Our case received regular rehabilitation, electrotherapy, and appropriate pain medication to aid in recovery. The initial recovery was satisfactory. However, post-operative ultrasonography (US) imaging revealed some local swelling and a small amount of effusion with enhanced bright spots Figure 2.

The patient showed good range of motion and was able to perform daily activities with ease six months post-surgery. However, from the 6 to 9 months, he has resumed mild jogging exercises and has reported experiencing localized pain and bruising in the heel region. The patient presented to the clinic 9 months after the initial operation, having engaged in marathon running without medical approval. Upon physical examination, ecchymosis and swelling were observed in the left heel and ankle. The Thompson test was positive, and a skin indentation was noted 5 cm from the heel.

### 2.2. Second operation

The patient underwent a revision surgery to reconstruct the AT using a hamstring tendon graft and endobutton pull out method. The incision wound had keloid tissue, which was revised before the surgery. The surgeon harvested two loop grafts, one of Semitendinosus tendon (ST) (25 cm in length) and the other of gracilis tendon (GT) (22 cm in length). After incising the peritenon tissue, the surgeon noticed necrotic tissue over the tendon repair site and poor tissue quality of the stump of the AT. About 2 cm of devitalized tissue from the end of the AT was removed. The surgical technique used in this case involved passing one limb of the ST through the AT stump transversely 4 cm distal to the end and re-routing it into the intrasubstance about 1 cm distal to the original entry point. The other limb of the ST was passed through the AT stump in the same way. The GT was passed through transversely, 6 cm distal to the end of the Achilles stump and sutured with #2 Ethibone using a baseball rolling suture method to the AT stump and ST graft. The distal parts of the two grafts were sutured together into a thick bundle loop graft and secured with an Arthrex ACL Tight-Rope endobutton.

**Figure 3:**
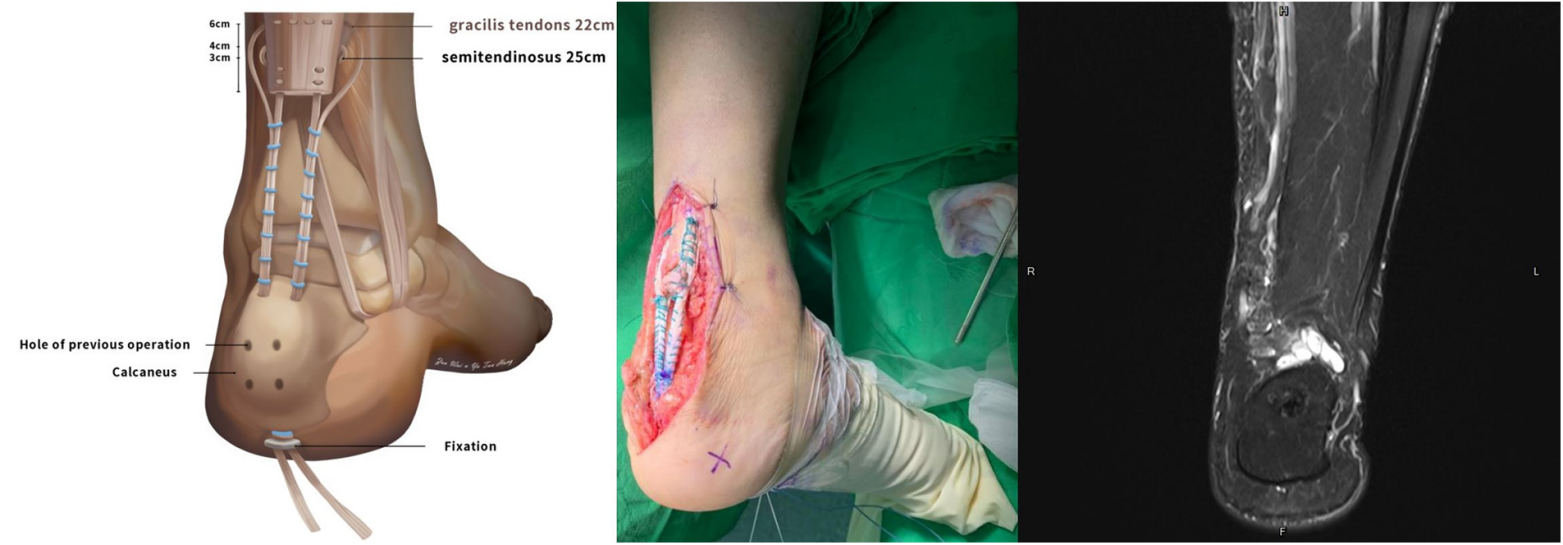
Illustration of the second operation (left), transosseous fixation (middle) and the resulted MRI image (right).

A guide pin was used to drill a calcaneal tunnel at the upper tip of the AT's footprint, angled 45 degrees towards the volar side of the calcaneal body. The tunnel was then fully lengthened using a 4.5 mm leading reamer. An 8 mm reamer was used to create a 3.5 cm depth graft tunnel, into which the endobutton CL loop was pulled, allowing the graft to merge into the tunnel. The tension of the AT and graft was assessed using the Thompson test. The wound was closed layer by layer to prevent skin tension, and a hemovac was used to drain tissue fluid. Temporary p-p splint fixation was applied, and the procedure was successfully completed without any immediate complications (Figure 3, left and middle).

At the 2-year follow-up, a postoperative radiograph and MRI PD-weighted imaging were conducted. The images showed consistent low signal intensity without any high signal or ruptured tendon fibers. The reconstructed tendons were healing well without any tendinosis, significant lengthening, or partial tearing. The T2-weighted coronal image showed a hypointense signal in the bone (Figure 3, right).

The post-surgery rehabilitation for AT involved a gradual recovery process. Initially using crutches for walking for about 3 months, the patient regained normal function in the affected limb within 4–5 months. Walking on tiptoe with the affected limb took up to 8 months. The rehabilitation program included personalized treatment with a physical therapist, incorporating manual therapy and muscle strength training. High-intensity exercises like marathon running and cycling were gradually introduced after 3 months, typically starting around 7 months post-surgery. By the end of the year, the patient achieved significant cycling endurance, indicating a successful recovery period of approximately 1 year.

## 3. Discussion

In this case study, a rare diagnosis of a partial AT tear was treated unsuccessfully due to the patient's early return to marathon running after six months of surgery. Subsequently, a complete rupture occurred nine months post-surgery, leading to a second revision surgery using the debatable ST and GT method. Fortunately, the revision surgery was successful, allowing the patient to resume marathon running and cycling without any complications.

During the early stages of an Achilles tendon (AT) injury, imaging is often unnecessary for diagnosis, as it can occasionally provide misleading or overly optimistic results. If the patient's clinical history points towards a complete tear, it should be regarded as such, even if the radiological report suggests a “partial tear.” While exceptions exist, it is typically prudent to err on the side of caution and refer the patient for further evaluation. In the specific case under consideration, surgical treatment was advised based on the initial physical examination, which indicated an almost complete tear (4).

Following surgical repair of a torn AT, the healing process involves distinct stages, including inflammation, tissue repair, and remodeling. During the early healing phase, acute scar tissue in the surgical area may exhibit magnetic resonance (MR) features similar to tendon rerupture, as both show T2-weighted hyperintensity. Additionally, due to tendon remodeling, the gap size on T2-weighted images may appear larger than anticipated. This highlights the importance of considering these factors when interpreting MR findings in post-operative cases of AT repair (5). This observation could potentially account for the hypointensity seen in the T2-weighted coronal image (Figure 3).

Reruptures and complications after AT repairs are infrequent, with reported rates ranging from 1.7% to 5.6%. A retrospective review in 2018 examined 423 patients who underwent surgical treatment and found a rerupture rate of 1% and an overall infection rate of 2.8%, with longer operative times showing a correlation. The risk of rerupture is commonly believed to be highest within the first 2 months after nonoperative treatment ends, although limited high-level evidence supports this claim. Some studies have indicated that the risk of rerupture is highest following the termination of cast or orthosis treatment in nonoperative protocols. Other factors such as type of immobilization, time to treatment, cause of rupture, gender, sports involvement, non-sports individuals, and immunosuppressive treatment did not demonstrate an increased risk of rerupture in certain studies (6).

Understanding the factors associated with the rerupture of the Achilles tendon post-surgery is crucial for optimizing patient outcomes. One critical aspect of this correlation lies in infection rates, both superficial and deep, occurring within the initial two years following surgery. A noteworthy connection exists between infection rates and the likelihood of Achilles tendon rerupture. Additionally, the various complications may arise in relation to patient-specific and surgery-specific factors. Furthermore, a similar trend has been observed in other orthopedic procedures, such as rotator cuff tendon repairs, where increased operative time has been statistically linked to a higher risk of rerupture. These findings underscore the significance of infection prevention strategies and efficient surgical techniques in the pursuit of successful Achilles tendon repair and overall improved patient care (7).

The revision surgery for the AT involves a comprehensive approach. Surgeons follow a systematic algorithm to address wound breakdown, infection, and rerupture. Infection clearance and debridement are crucial before evaluating tendon quality. Primary repair is performed if no tendon defect is present, with attention to protecting the posterior soft tissue. Tendon transfers are considered for weakened repairs, while procedures like gastrocnemius aponeurosis lengthening or turndown techniques are used when reapproximation is not possible. Autografts or allografts may be utilized in conjunction with these methods for complex revision cases involving large tendon deficits (8).

In recent years, there has been a growing interest in utilizing autografts and transfers as augmentation options for chronic or revisional AT ruptures. Maffulli et al. (2014) introduced a minimally invasive free semitendinosus graft technique for reconstructing chronic deficits, which resulted in good to excellent outcomes in 93% of the 28 patients treated, with 57% returning to their preinjury level of sport. Similarly, Patil et al. (2014) achieved satisfactory functional outcomes and no reruptures in 35 chronic rupture cases by employing an open technique that harvested the semitendinosus tendon (9).

Jiang et al. in 2019 (10), reported surgeries on seven patients (6 males, 1 female) with AT rupture using autografts from the ST and GT. The patients were followed for an average of 31.3 months. Six out of the seven patients expressed a desire to return to sports, and half of them were able to do so successfully. All patients reported positive postoperative clinical outcomes. Notably, our study yielded promising results even with a tendon gap of 4 cm (that became 6 cm after the resection), and the patient was able to resume athletic activities after one year.

In their 2022 study, Nilsson et al. examined 22 patients (13 males, 9 females) with a median age of 64 who underwent surgical reconstruction of the AT using an endoscopically assisted technique with a ST autograft. The patients were evaluated at the 12-month mark, leading to the conclusion that this approach is effective in treating chronic AT ruptures and reruptures. The technique yields satisfactory outcomes, particularly for patients with distal ruptures or large tendon defects, as it successfully restores heel-rise height. Overall, this technique presents a viable option for AT reconstruction (11).

The use of ST autograft augmentation has been a subject of debate in some studies. A retrospective study examined 58 patients who received ST autograft augmentation at Helsinki University Hospital. Complications were observed in 24% of cases, primarily infections that occurred approximately 62 days post-surgery. Some patients required additional procedures to address issues like skin edge necrosis, deep infection, hematoma formation, or repeated rupture. As a result, the authors discourage the use of autologous ST grafts for AT reconstruction at their institution. They suggest considering alternative methods like flexor hallucis longus tendon transfer. It should be noted that there is limited evidence supporting the use of semitendinosus grafts for primary repair of acute AT tears and augmentation techniques are associated with increased risks of complications. Hamstring tendons have shown success in reconstructing chronic tears, but the avascular nature of semitendinosus grafts may heighten infection risks. Clinics with higher complication rates may attribute them to factors such as lengthy incisions, extensive tendon exposure, and aggressive debridement. Utilizing less invasive techniques could help minimize wound healing complications (12).

Exploring alternative tendon options before surgery is a viable consideration. It's worth noting that this study's limitations include the absence of stronger recommendations to prevent rerupture. In an animal study (13), researchers examined the effects of sodium hyaluronate (HA) and chondroitin sulfate (CS) combination on tendon healing and peritendinous adhesion. Rats receiving higher doses of HA + CS displayed reduced inflammation, less adhesion, and significantly stronger tendons compared to controls. This suggests that HA + CS treatment can effectively prevent post-tendon surgery adhesion, potentially contributing to quicker rehabilitation when considering alternative treatments.

The patient expressed deep gratitude towards the doctors at Tri-Service General Hospital for their invaluable assistance. The past three years had been an arduous journey for the patient. Reflecting on the experience, the patient acknowledged that avoiding heel steroid injections and refraining from rushing into marathons after the initial operation would have been wise decisions. Nevertheless, the patient found great satisfaction in the second operation. Aside from experiencing some soreness and temporary loss of strength, no significant issues arose. The patient was delighted to resume long-distance running and cycling without encountering any major obstacles. The patient extended heartfelt thanks for the surgical intervention and the provided treatment.

## Conclusion

In differential diagnosis, partial tears of the AT should be considered as an alternative diagnosis in the patients with heel pain. A proper post-surgical rehabilitation process is crucial for AT surgeries. When AT surgery proves unsuccessful, one can explore the option of using grafts from the gracilis and semitendinosus tendons for reconstruction in cases of reruptures.

## Data Availability

The original contributions presented in the study are included in the article/Supplementary Material, further inquiries can be directed to the corresponding authors.
